# Pathologic stage dictates survival after neoadjuvant radiation for rectal cancer

**DOI:** 10.18632/oncotarget.26262

**Published:** 2018-10-26

**Authors:** Daniel Delitto, Tyler J. Loftus, Atif Iqbal

**Affiliations:** Department of Surgery, University of Florida College of Medicine, Gainesville, FL, USA

**Keywords:** rectal cancer, pathologic stage, clinical stage, prognosis, survival

With the advent of large-scale multicenter data repositories, prognostic data has evolved, causing paradigm shifts in therapeutic approaches and impacting informed discussions with patients and their families. This is especially true with the implementation of neoadjuvant treatment protocols in oncology. For patients with rectal cancer, neoadjuvant chemotherapy and radiotherapy are the standard of care for patients with stage II-III disease, often downstaging the disease. Historically, prognosis in this patient population has been controversial, in part due to variation in responses to neoadjuvant therapy. While it is relatively well accepted that a pathologic response to neoadjuvant therapy results in a favorable prognosis, data regarding the magnitude of this effect are conflicting [1-3]. Specifically, data regarding the relative importance of a patient’s presenting clinical stage versus postoperative pathologic stage have been lacking. To address this, we queried the National Cancer Database (NCDB), a dataset large enough to provide insight into a straightforward prognostic question [4]. Methods and major findings are summarized in Figure 1.

**Figure 1:**
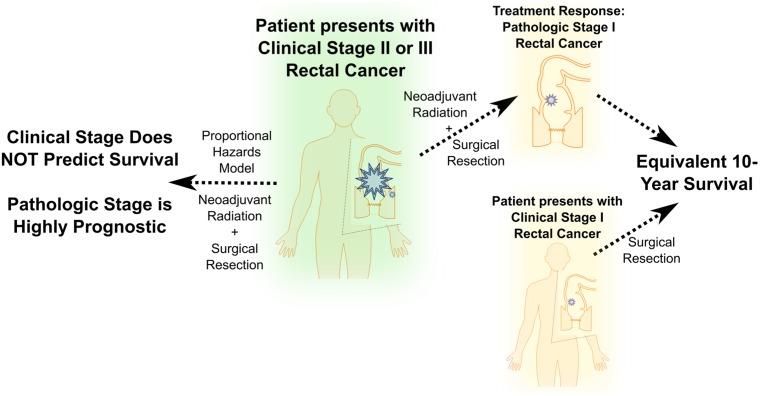
Major findings from an analysis of National Cancer Data Base subjects presenting with clinical stage II or III rectal cancer

Although the NCDB is subject to variability in local practices for staging and neoadjuvant therapy protocols, this heterogeneity also improves generalizability of our findings. We tried to overcome this by strict inclusion/exclusion criteria intended to focus the study on pertinent patient populations, which may have introduced a selection bias. We excluded patients with local excision and those with positive surgical margins, as these patients are at increased risk for local recurrence. Provision of adjuvant radiotherapy was rare. As the indications for adjuvant radiotherapy as well as its impact on outcomes are unclear, these patients were also excluded. Although these exclusions seem appropriate for a study assessing the prognostic value of clinical versus pathologic stage for patients with rectal cancer receiving neoadjuvant therapy, careful statistical analysis remains necessary to ensure the reliability of the results. We employed inverse probability weighting by generating predicted probabilities of inclusion for each subject, including subjects excluded from the main analysis, and applying heavier statistical weight to subjects that were under-represented in the main analysis. When applying the inverse probability weights, pathologic staging yielded highly accurate prognostic information compared to clinical staging, similar to the main analysis.

Despite these limitations, the resultant data were definitive. For a patient undergoing neoadjuvant radiation with stage II-III rectal cancer, the presenting clinical stage was largely irrelevant in predicting prognosis. Conversely, the postoperative pathologic stage was highly prognostic, to the point that stage III patients downstaged to ypT1N0 disease demonstrated equivalent outcomes to patients presenting with early stage cT1N0 disease proceeding directly to surgery. Our results are highly relevant to discussions with patients presenting with locoregional disease, as their prognosis ultimately hinges on their response to neoadjuvant radiation, and may be significantly more favorable.

Several important questions remain. The accuracy of clinical staging is particularly relevant to our conclusions. This is commonly performed with endorectal ultrasound, magnetic resonance imaging (MRI), or both. The ideal approach remains controversial although the improving resolution of MRI and reproducibility is leading to increasing national reliance on MRI [5]. At this time, the NCDB does not allow for direct comparison of these methods. Similarly, molecular profiling is a promising adjunct to current staging methods, and its potential impact on prognostic accuracy is an area of ongoing investigation [6, 7]. Finally, the optimal interval between completion of neoadjuvant therapy and surgical resection also remains unclear [8, 9]. Future investigations targeting these knowledge gaps may continue to improve prognostic accuracy for patients with rectal cancer as therapeutic approaches continue to evolve.

## References

[1] Quah HM (2008). Cancer.

[2] Kim CH (2016). J Surg Oncol.

[3] Fokas E (2015). Cancer.

[4] Delitto D (2018). J Natl Cancer Inst.

[5] Rengo M (2017). Oncotarget.

[6] Hiyoshi Y (2017). Oncotarget.

[7] Sun M (2017). Oncotarget.

[8] de Campos-Lobato LF (2011). J Gastrointest Surg.

[9] Li YH (2017). Oncotarget.

